# A critical appraisal of the status and hydrogeochemical characteristics of freshwater springs in Kashmir Valley

**DOI:** 10.1038/s41598-022-09906-2

**Published:** 2022-04-06

**Authors:** Sami Ullah Bhat, Shahid Ahmad Dar, Aadil Hamid

**Affiliations:** Department of Environmental Science, University of Kashmir, Srinagar, J&K 190006 India

**Keywords:** Environmental sciences, Hydrology

## Abstract

With growing water scarcity, jeopardized by climate change, springs are likely to perform a vital role in meeting the domestic water demand in future. This paper examines the water quality status of Kashmir valley springs in relation to their geographical location, regional hydrogeological conditions, anthropogenic activities and climate change. We analyzed data for 258 springs from the whole Kashmir valley using water quality index (WQI) and geographic information system techniques. WQI ranged from 23 (excellent water) to 537 (water unsuitable for drinking). The WQI indicated that 39.5% of the springs had excellent waters, 47.7% had good water, 5% had poor water, 1.6% had very-poor water, and 6.2% of the springs had water unfit for drinking purposes. The Piper diagram identified Ca–Mg–HCO_3,_ Ca–Mg–SO_4,_ and Na–HCO_3_ as the most predominant hydro-chemical facies, whereas Gibbs diagram revealed that the water of springs in the study region is mainly controlled by rock weathering dominance. The results of the study offer inputs about the water quality to be used by the concerned departments and agencies at a bigger scale for drinking purposes. Our findings therefore suggest that springs which are in thousands in Kashmir landscape have the potential to offer viable solution to the rising drinking water demand and therefore merit an attention for their protection and management.

## Introduction

The scarcity of water in many regions of the world has become an unpleasant reality^1^. Freshwater has become a stressed resource and its availability has become increasingly limited^2,3^. With the fast pace of urbanization and industrialization, climate change and rising temperatures, and a marked decrease in rainfall, the problem of water scarcity is being gradually felt throughout the globe^4^. The water economy is under massive stress and supplying safe drinking water to a growing global population is one of the major challenges for water resource managers^5^. River and stream systems may not be able to meet the future water demands for industrial, agricultural, and domestic uses due to unscientific and improper use of water and rising pollution^6,7^ which has led to huge scarcity of water supply in many regions of the world^8^. Due to inadequate quality or unavailability of freshwater, demand for drinking water has increased over the years, especially in densely populated, arid, and semi-areas regions of the world^9^. As a result, 40% and 20% of the world population is now facing severe and high-water stress respectively^10^. Additionally, the increasing demands for water resources has exaggerated conflicts between nations, thus increasing the probability of a third world war. Water from freshwater springs may help alleviate this situation. Worldwide, 1/4th of the consumption of water relies on underground sources, which contributes 36% to drinking, 27% to industrial, and 42% to irrigation^11^. During the last few years freshwater springs have gained increased status and recognition because of the vital role they perform in meeting the growing demands for drinking water^12^. They have been the source of freshwater supplies for human populations around the world, guaranteeing domestic water of rural and urban populations, supporting socio-economic progress, and sustaining environmental balance.

India has approximately 5 million springs, including nearly 3 million in the Indian Himalayan Region (IHR) alone^13^. These springs are a source of freshwater for over 200 million people. An estimated 80–90% of the population in the Himalayas depends on springs for their daily use^14^. The existence of springs is not restricted to rocks of any specific type or age group or any particular topographic or geological setting. They occur wherever groundwater emerges naturally from soil, sediment, or rock into a water body or onto the earth’s surface^15^. Therefore, the variety of springs is suggestive of the wide range of hydrologic and geologic settings which lead to their existence^16^. Water quality of freshwater springs varies both in time and space based on rock formations, source of aquifers, mineral dissolution, ion exchange, intermingling together with pollutants^17^. Utilization of springs whether indirect or direct provides numerous benefits to human civilizations, but this resource has been associated with substantial costs to the environment, including biodiversity loss, and deterioration of water quality^18^. The quality of water is regularly declining from the effects of overutilization^19^, mixing of pollutants^20^, land-use-land-cover changes, and mining activities^21^. As a result, spring water resources are severely diminishing in quality and quantity in several parts of the world, especially in arid and semi-arid regions^22^. Despite their critical importance, springs have received little recognition in terms of management and conservation^23^. Over the past few decades, freshwater springs have been declining in quantity and quality throughout the world due to overexploitation, human population growth, declining precipitation, and climate change^24^.

Throughout the globe, springs have remained data deficient and have not relatively received much attention as compared to other systems like lakes, wetlands, rivers, streams. This is also true for the Kashmir valley wherein most of the research is focused on lakes and wetlands^25,26^ and there are also comparatively a large number of workers/researchers and institution working on lakes and wetlands. In Kashmir Himalaya, springs play an important role in supplying drinking water, especially in rural settings besides role in agriculture, trout fisheries and other ecosystem services and desired uses^27,28^. During the last few decades, the freshwater springs of the Kashmir valley have been under increasing risk of depletion due to anthropogenic activities and changing climate^29^. Large scale land use changes^30,31^, massive deforestation in catchment areas, and infrastructural development have largely disrupted the hillslope hydrology in the Kashmir valley. This has led to depletion, flow reduction, and drying up of natural freshwater springs. Despite the huge importance of springs, little attention has been paid to their management and conservation^32^.

To date, there are only few publications on spring water in Kashmir valley. On one hand there seems to be information available on hydrochemistry of springs^33,34^ and hydro-geochemistry^35^ but on the other hand ecosystem perspective of springs is missing wherein there are very little works who have comprehensively documented crenic biodiversity^36,37^ in some select areas. In the above noted scenario, the present study takes comprehensive account of water quality and hydrochemistry of springs in Kashmir valley using robust methods in GIS environment. The main objectives of this work are to (a) assess the overall water quality of springs in Kashmir valley (b) evaluate the chemical relationships governing the most predominant water in the study area, and (c) to identify the underlying processes governing the quality of spring water. It is anticipated that the study will benefit the planners and water resources managers in the region to have comprehensive policy regarding use and management of freshwater springs.

## Results

### Water quality

**Figure 1:**
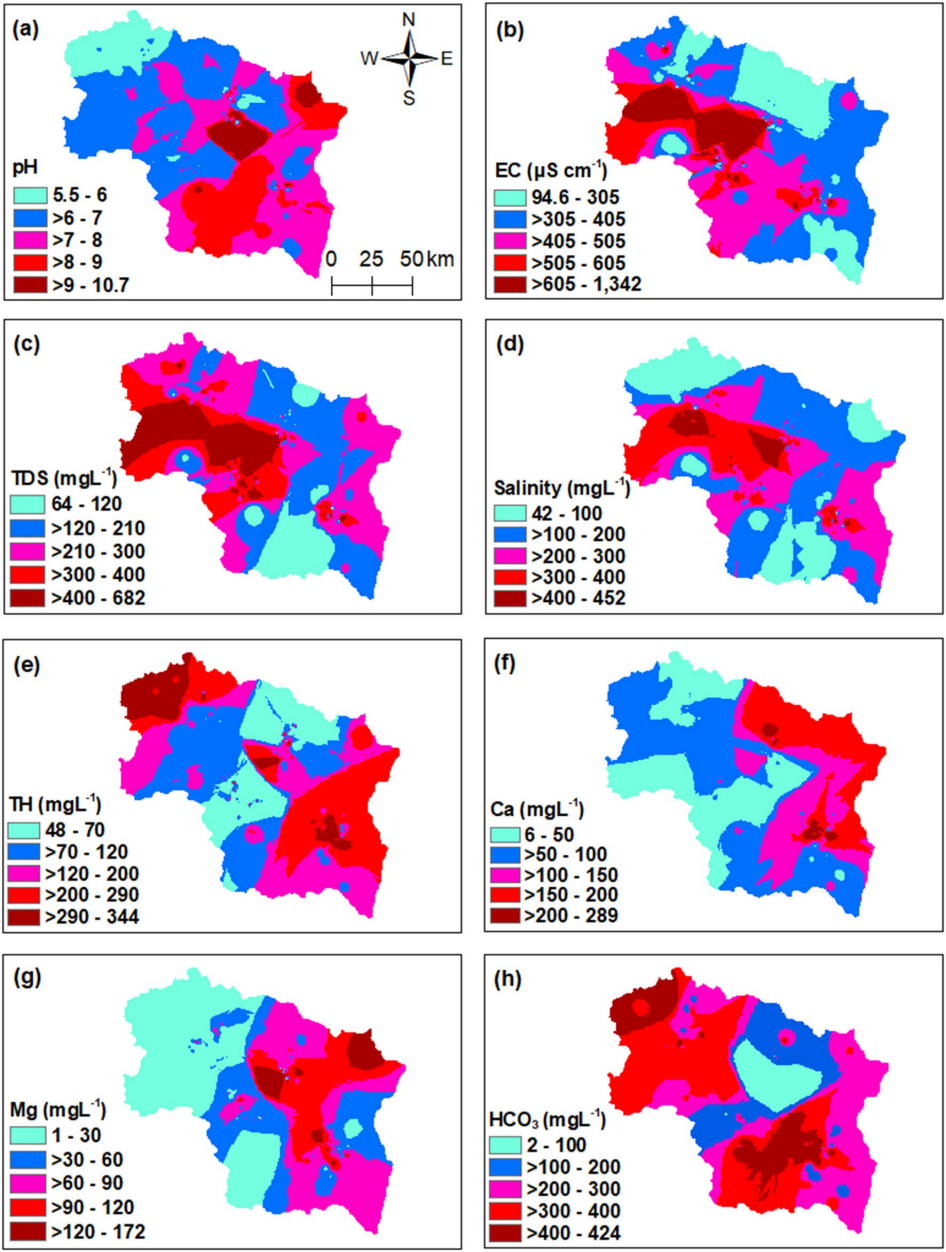
Spatial distribution of (**a**) pH, (**b**) electrical conductivity (µS cm^−1^), (**c**) total dissolved solids (mg L^−1^), (**d**) salinity (mg L^−1^), (**e**) total hardness (mg L^−1^), (**f**) calcium (mg L^−1^), (**g**) magnesium (mg L^−1^), and (**h**) bicarbonate alkalinity (mg L^−1^) of freshwater springs in Kashmir valley. The figure was generated using spatial analyst module and Natural Neighbor interpolation in ArcGIS version 10.4.1. (https://www.esri.com/en-us/arcgis/products/arcgis-pro/).

**Figure 2:**
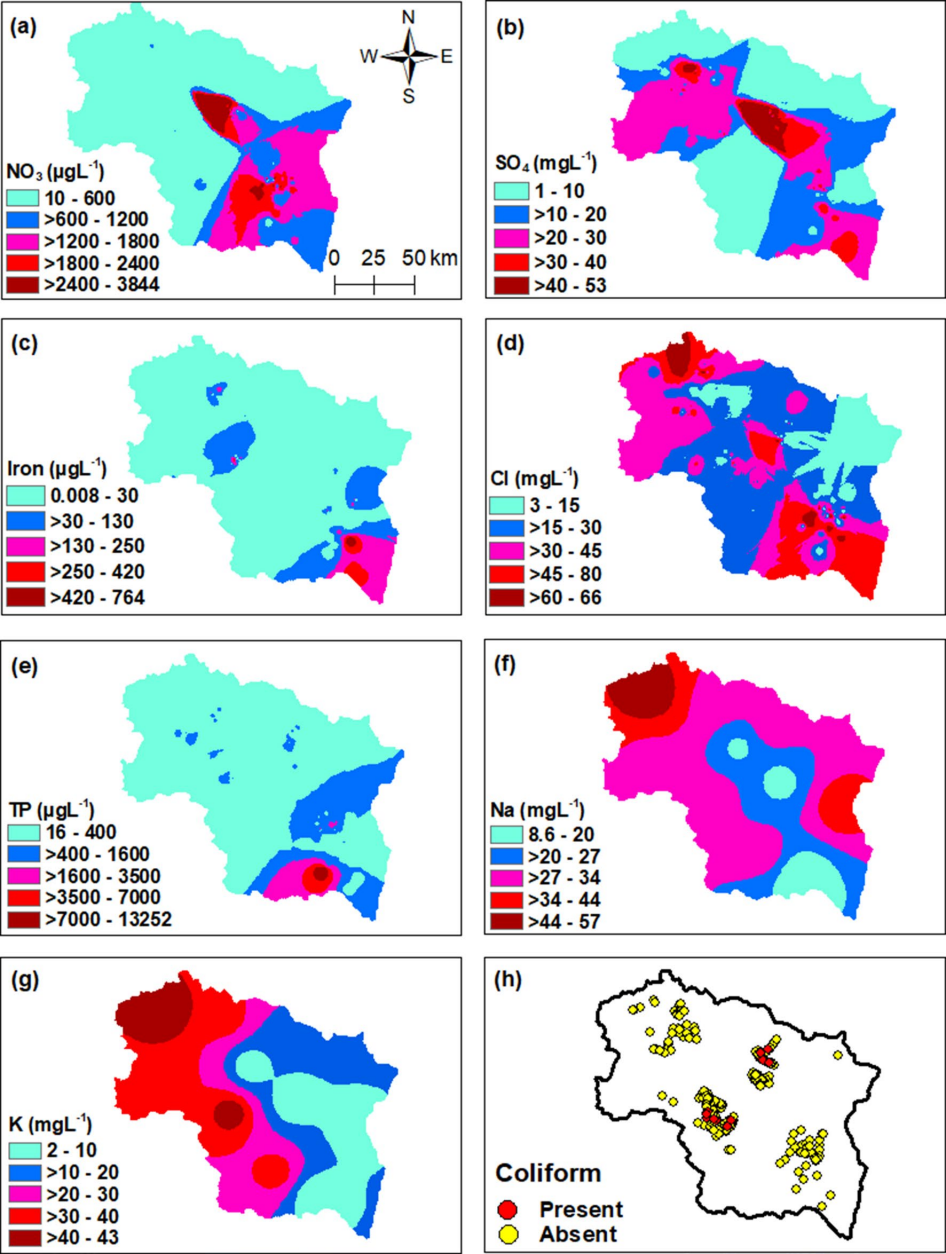
Spatial distribution of (**a**) nitrate (µg L^−1^), (**b**) sulphate (mg L^−1^), (**c**) iron (µg L^−1^), (**d**) chloride (mg L^−1^), (**e**) total phosphorus (µg L^−1^), (**f**) sodium (mg L^−1^), (**g**) potassium (mg L^−1^), and (**h**) coliform of freshwater springs in Kashmir valley. The figure was generated using spatial analyst module and Natural Neighbor interpolation in ArcGIS version 10.4.1. (https://www.esri.com/en-us/arcgis/products/arcgis-pro/).

Evaluating the quality of spring water is essential for determining its fitness for drinking purposes. The various physical, chemical, and biological (coliform) characteristics of spring waters were related with the standards of drinking water quality set by WHO^38^. The concentration values of various water quality parameters are given in Table 1. The pH is an important characteristic that describes the acidity and alkalinity of water samples. The chemical characteristics show that the spring water samples are acidic to alkaline in nature with a pH value ranging from 5.5 to 11. The spatial distribution of pH in the study area is shown in Fig. 1a. Among the investigated samples, 95% of the samples had pH values within the desirable limits, 3% had pH values in the acidic range, and 2% had pH values above the permissible limits^38^. Electrical conductivity (EC), ranged from 90 to 2710 µS cm^−1^. The spatial distribution of EC in the study area is shown in Fig. 1b. It was found that 99.6% of the samples had EC values within the permissible limits and thus only 0.4% had EC values beyond the permissible limits set by WHO^38^. The concentration of total dissolved solids (TDS) signifying the various types of dissolved minerals present in the water samples varied between 64–682 mg L^−1^. The spatial distribution of TDS in the study area is shown in Fig. 1c. About 16.3% of the samples show TDS contents above the WHO desirable standard value^38^. Davis and De Wiest^39^ classified TDS values into four categories (1) TDS < 500 mg L^−1^ as desirable for drinking, (2) TDS between 500–1000 mg L^−1^ as permissible for drinking, (3) TDS between 1000–3000 mg L^−1^ as useful for irrigation, and (4) TDS > 3000 mg L^−1^ as unfit for drinking and irrigation. According to this classification, about 84% of the samples in the study area fall in desirable category and 16% of the samples were within permissible limits (Supplementary Table S1). Salinity in the study area varied from 42 to 452 mg L^−1^ (Fig. 1d). Water quality evaluation of the springs in the study area also indicated that the waters are soft to very hard. The concentration of total hardness (TH) generally caused by the compounds of calcium, magnesium, and other metals in the study area ranged from 48 to 344 mg L^−1^ (Fig. 1e), well below the maximum permissible limit of 500 mg L^−1^ set by WHO^38^. Furthermore, 25% of the samples were hard and 4% very hard, following the classification by Sawyer and McCartly^40^ (Supplementary Table S1). The concentration of calcium in the study area varied between 6–289 mg L^−1^. The spatial distribution of calcium in the study area is shown in Fig. 1f. We found that 4% of the samples had concentrations above the permissible limits set by WHO^38^. The concentration of magnesium varied between 1–172 mg L^−1^ (Fig. 1g). The concentration of magnesium in 60.5% of the samples was within the desirable limits, the concentration in 38.8% samples was within the permissible limits, and 0.8% samples had concentrations above the permissible limits set forth by WHO^38^. Bicarbonate alkalinity in the study area ranged from 2 to 424 mg L^−1^ (Fig. 1h). The concentration of nitrate in the spring water samples varied between 10–3844 µg L^−1^ (Fig. 2a) and was within the desirable limits set by WHO^38^. The concentration of the SO_4_^2−^ varied between 1–53 mg L^−1^. The spatial distribution of SO_4_^2−^ is shown in Fig. 2b. SO_4_^2−^ concentrations are all within the desirable limit of 200 mg L^−1^ set by WHO^38^. Iron concentrations in the study area ranged from 0.008 to 764 µg L^−1^ (Fig. 2c). Chloride concentrations varied between 3–66 mg L^−1^ (Fig. 2d), well within the desirable limits set by WHO^38^. The concentration of total phosphorus (TP) in the study area ranged from 16 to 13,252 µg L^−1^. The higher concentrations were found in the south of the Kashmir valley (Fig. 2e). Based on concentration of TP, only 66.7% of the samples were suitable for drinking purposes and 33.3% of the samples were not suitable for drinking purposes as per the Environmental Quality Standards for Surface Water of the People’s Republic of China (GB3838-2002)^41^ (Supplementary Table S1). The concentration of sodium varied from 8.6 to 57 mg L^−1^ (Fig. 2f) and potassium varied from 2 to 43 mg L^−1^ (Fig. 2g). In the study area, the presence of Coliform bacteria occurred in 5.4% of the investigated samples and the value ranged from 3 to 28/100 ml (Fig. 2h).

**Table 1:** Comparison of water quality of springs in Kashmir valley with WHO, (2017) for drinking purposes.

S. no	WQ parameters	Concentration of water quality parameters in study area	WHO, 2017	
			Highest desirable limit	Maximum permissible limit
1	pH	5.5–11	7.0	8.5
2	EC (µS cm^−1^)	90–2710	–	1500
3	TDS (mg L^−1^)	64–682	500	1500
4	Salinity (mg L^−1^)	42–452	–	–
5	TH as CaCO_3_ (mgL^−1^)	48–344	100	500
6	Ca^2+^ (mg L^−1^)	6–289	75	200
7	Mg^2+^ (mg L^−1^)	1–150	30	150
8	HCO_3_ (mg L^−1^)	2–424	–	–
9	NO_3_^−^ (µg L^−1^)	10–3844	45	–
10	SO_4_^2−^ (mg L^−1^)	1–53	200	400
11	Iron (µg L^−1^)	0.008–764	–	–
12	Cl^−^ (mg L^−1^)	3–66	200	600
13	Total phosphorus (µg L^−1^)	16–13,252	–	–
14	Na^+^ (mg L^−1^)	8.6–57	–	200
15	K^+^ (mg L^−1^)	2–43	–	–
16	Coliform (per 100 mL)	3–28	0	–

### Water quality index (WQI)

WQI ranged from 23 (excellent water) to 537 (water unsuitable for drinking) (Supplementary Table S2). The WQI indicates that 87% of the samples have waters between good to excellent water and are fit for drinking purposes without any treatment. Approximately 7% of the samples have water quality ranging from poor to very poor and require minimal treatments before being used for drinking purposes. 6.2% of the samples have water unsuitable for drinking purposes.

### Principal component analysis (PCA)

PCA was performed on water quality parameters with 258 sampling sites to identify variation in water quality. The variable loadings and variance (%) for the four components derived from the dataset is given in Supplementary Table S3. This analysis led to the cumulative explanation of 31%, 49%, 59%, and 67% of the variance.

### Hydro-chemical facies

**Figure 3:**
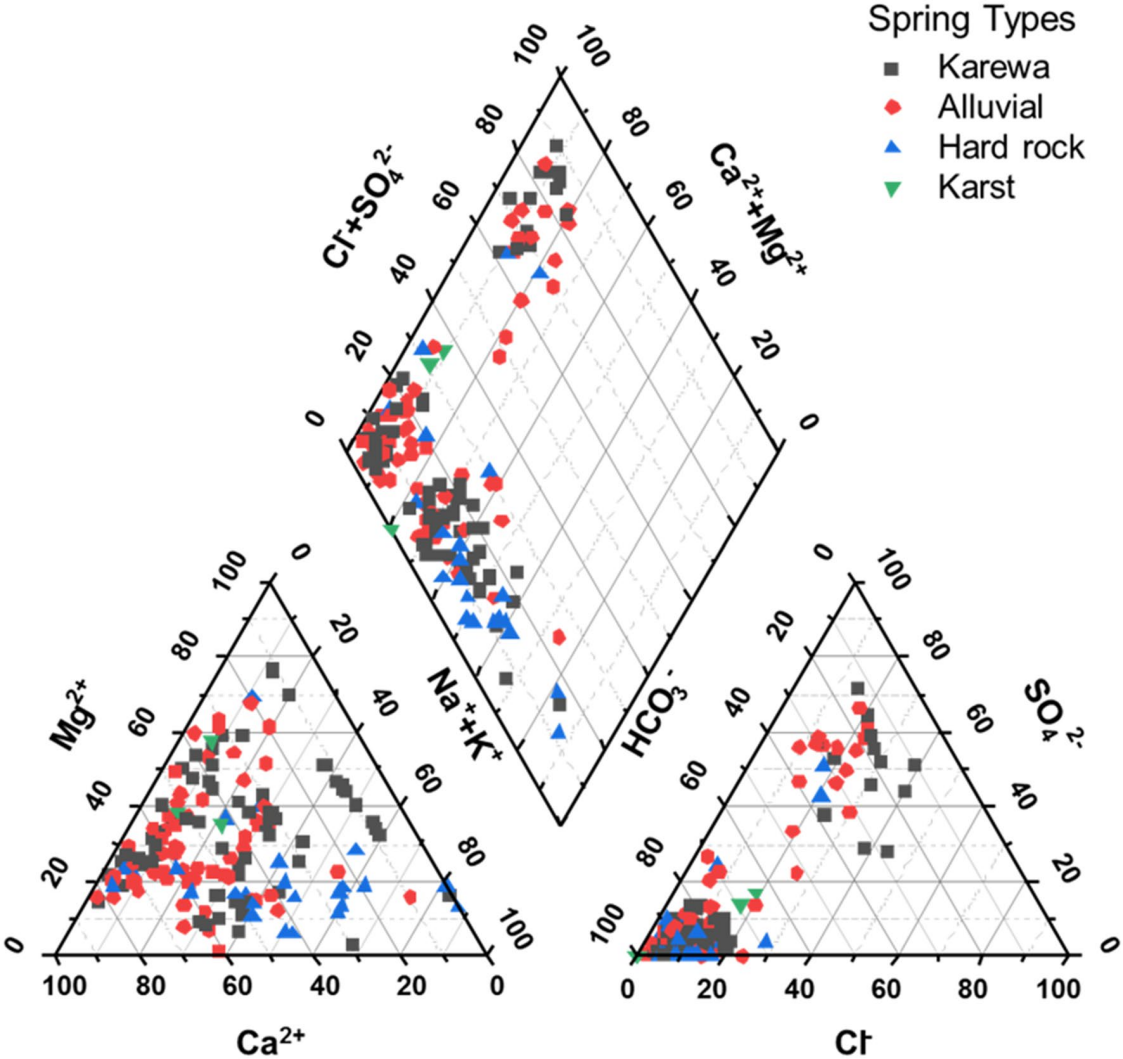
Piper trilinear diagram of the major cations and anions of the springs. This figure was plotted using R programming for statistical computing (https://cran.r-project.org/bin/windows/base/).

**Figure 4:**
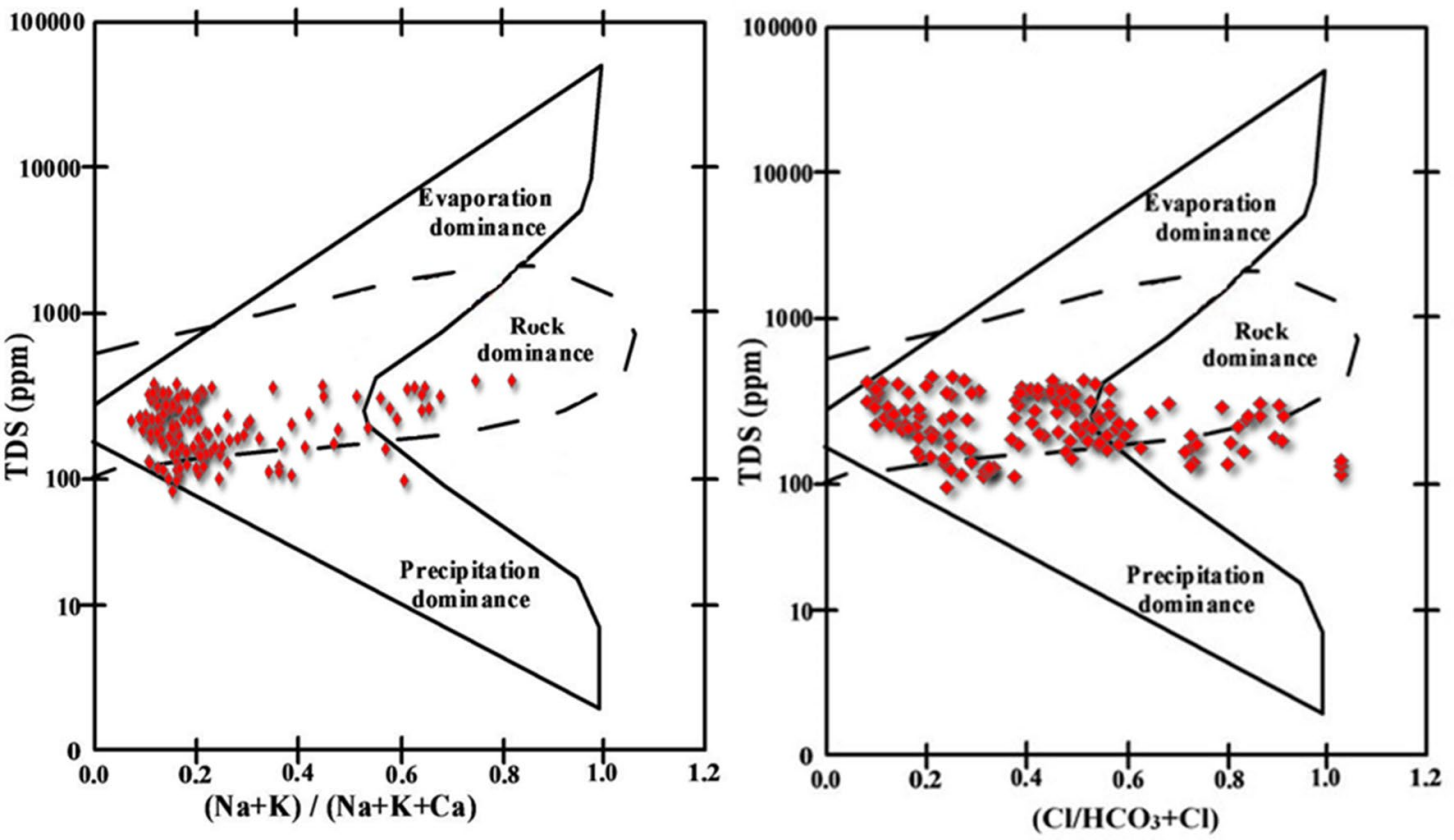
Gibbs diagram showing TDS vs Na^+^ + K^+^/Na^+^ + K^+^ + Ca^2+^ and Cl^−^/HCO_3_^−^ + Cl^−^ for evaluating factors controlling spring water chemistry. The figure was generated using Microsoft Excel 2016 (https://support.microsoft.com/en-us/office).

The major cations in the spring water samples were in the order of Ca^2+^ > Mg^2+^ > Na^+^ > K^+^, with contribution of 48%, 31%, 13%, and 8% respectively. The most abundant anions were HCO_3_^-^, Cl^−^ and SO_4_^2−^, with contribution of 81%, 11%, and 8% respectively. Piper trilinear diagram showed that most spring water samples (74%) fall into the left quadrant of the central diamond plot indicating Ca–Mg–HCO_3_ waters. Spring water samples (20%) in the top quadrant of the diamond plot are Ca–Mg–SO_4_ waters, while samples in the bottom quadrant of are Na–HCO_3_ waters (Fig. 3). Since, Ca–Mg–HCO_3_, Ca–Mg–SO_4_, and Na–HCO_3_ are the most common hydro-chemical facies, it is likely that lithology and human activities have played an important role in controlling the spring water chemistry in the Kashmir valley. The Gibbs diagram indicating the ratio Na + K/Na + K + Ca and Cl/HCO_3_ + Cl as a function of TDS revealed that the rock weathering as the major factor, with precipitation as a minor factor, thus controlling the water chemistry in the study area (Fig. 4).

## Discussion

The analysis of water quality of springs in Kashmir valley revealed that majority (~ 87%) of the investigated springs can be used for drinking purposes without any treatment. As we look over the huge datasets with regard to water quality of springs in the Kashmir valley, it is apparent that they have reasonably the large potential to meet the rising demands of the zooming population in the region. Most of the investigated springs have water in alkaline scale while water quality of few springs in Kupwara district displayed slightly acidic character. The possibility of acidic nature may be of local origin arising due to release of organic acids and high carbon dioxide content whereas the high alkaline nature along the central and south of study area is related to the limestone-rich lithology of the Kashmir valley^42^. Comparatively high EC values in Kupwara and Baramulla districts indicate the potential impact of inorganic fertilizers and inputs of domestic sewage from adjoining catchment areas^43^. Salinity is not a major concern in the study area and is related to the TDS values caused by the dissolution of minerals gypsum, carbonates, and sulphate salts. The predominant source of bicarbonates (alkalinity), total hardness, and calcium ion in the study area is the carbonate lithology which indicates the intense dissolution and chemical weathering of calcite minerals^35^. The magnesium values indicate contribution through dissolution of pyroxenes, dolomites, and amphiboles^44^. The possible sources of sulfates and nitrates in the study area reveal intense leaching and surface runoff from soils and agricultural fields, leakages from septic tanks, surface drains, and domestic sewage^32^. The sources of chloride in the study area are related to the dissolution of soil salts, finer detrital sediments comprising silt/sandy-silt/clay and sandy clay. Fairly low chloride concentrations reveal low background levels from the lithological foundations in the area. The sodium and potassium content also reflect the major influence on their origin by the lithology of the catchment area. The sodium ions in spring water owe their origin from the interaction of meteoric water with primary silicates, alumino-silicates and clay minerals. However, some enrichment from anthropogenic activities and water bodies (lakes/wetlands, and paddy fields) particularly in low-lying areas cannot be overlooked. The increasing concentration of sodium and potassium along the Kupwara is an indication of increasing human activities. Further, the amendment of soils in the horticultural and agricultural fields are the possible secondary sources of increased potassium concentration comparative to normal background levels along the central and south Kashmir. According to WHO^38^, coliform should not be present in any of the samples for drinking purposes, therefore, the presence of coliform bacteria in some springs of Ganderbal and Budgam district indicates the contamination of aquifers by septic tanks. This is further supported by the fact that Budgam and Ganderbal districts don’t have any sewage treatment plants and all of the fecal matter is buried underground. WQI indicated that the majority of the springs have excellent to good water, whereas few springs have very poor-quality waters.

The PC1 explained 31.08% of the total variance and had strong loading of TH, Ca^2+^, Mg^2+^, SO_4_^2−^, NO_3_–N, Cl^−^, and TP. The PC2 explained 18.08% of the total variance and had strong loading of EC, TDS, and Salinity. The PC3 explained 9.2% of the total variance and had strong positive loadings of pH, bicarbonates, and SO_4_^2−^. The PC4 explained 8.1% of total variance with strong positive loading for coliform concentration.

The piper trilinear diagram results revealed that most (76%) of the spring water samples from the study area are dominated by Ca–Mg–HCO_3_ typical of shallow groundwater derived from limestone, dolomite, gypsum, and carbonate weathering/dissolution^45^. The second dominant water type (20%) based on hydro-chemical facies belonged to Ca-Mg-SO_4_, typical of gypsum groundwater derived from calcite precipitation or calcium removal ion exchange process and carbonate–silicate weathering and dissolution^46^. The third water type (6%) belonged to Na-HCO_3_ group, typical of deep groundwater with high influence of ion-exchange process weathering and dissolution of silicate and evaporating rocks^47^. Gibbs plot revealed that the rock-water interaction is the main driving force, with the precipitation being minor force controlling hydro-chemistry of the studied springs spread across the Kashmir valley.

Water quality maps provide insights on which springs are vulnerable due to geogenic or anthropogenic activities. The GIS and WQI clearly recognized the water quality of springs with much robustness and would therefore help planning for conservation and management of these important water resources. The datasets arising out of this research work will provide the base for various stakeholders like local communities, policy makers, governments, researchers and students to draw the conclusion and recommendations thereof with regard to spring monitoring, restoration and management.

### Spring-shed management

Spring-shed management has arisen as a ray of confidence to alleviate depletion of spring water and is gradually spreading to spring-scapes throughout the globe. A spring-shed approach comprises a combination of aquifer, watershed, and landscape as elements of management. Appreciation of springs as groundwater dependent ecosystems is vital to their protection and management, because abstraction and consumptive use as well as changing land-use patterns that affect quality of aquifers, are key threats to the veracity of spring ecosystems. The complete hydrogeological mapping, data monitoring mechanisms, measuring hydrological and socio-economic impacts, social, gender, and governance aspects, conceptual hydrogeological layout of spring-sheds, classification of spring types and recharge areas, protocol and implementation of spring-shed management are the key steps that need to be followed for management of spring-sheds. Interestingly shift from watershed to spring-shed and spring study to rejuvenation and revival of springs has gained its momentum in India as witnessed from some studies and discussions on policy initiatives and documents on springs in the recent past^48–50^. But success of such concepts has not been independently and sufficiently demonstrated on a larger scale. In particular, 116 springs have been subjected to rehabilitation, out of a total of 3560 springs that have been georeferenced among 18 watersheds in the Indo-Himalayan states of Arunachal Pradesh, Himachal Pradesh, Jammu and Kashmir, Nagaland, Sikkim, and Uttarakhand. Nevertheless, efforts from various individual education and research institutions, community level, NGOs and few Govt. departments are in swing to further the cause of spring research across IHR. The States which have dominated the spring research in IHR include Jammu and Kashmir, Sikkim, and Uttarakhand.

### Climate change and role of springs in Kashmir valley

Assessment and monitoring of hydro-geochemical and physiochemical properties of natural springs is crucial in the context of climate change scenarios^51^. Changing climate and growing human population have jeopardized the water resource base and availability^52^. Climate change impacts have altered the timing and magnitude of potential recharge through changing precipitation patterns, water availability, floods, and drought^53^. Like many regions of the world, Kashmir valley is highly vulnerable to impacts of changing climate^54^.

In the Kashmir region, it has been observed that from 1980 to 2016, the average temperature has increased by 0.8 °C while as the precipitation has significantly decreased^55^. In Kashmir valley, the changing climate may influence the recharge of springs in numerous ways. The climate change affects recharge by reducing the amount of soil infiltration and percolation of water to deeper surfaces. Furthermore, the rising temperatures in valley increase the evaporation over land surfaces thereby limiting the amount of water to replenish underground aquifers. Although the level of groundwater in most of the plains does not seem to have been adversely affected, but on an average the level of groundwater in Karewas and upper areas has decreased by one-third. Studies indicate that the instances of springs drying is increasing in the Kashmir valley^56^. This has been attributed to glacier retreat, pollution, blocking of feeding channels, and forest denudation. An important finding is that future changes in recharge of spring water are not only ruled by the anticipated vagaries in precipitation but also by other hydrological processes such as snowmelt and evapotranspiration. Further the studies indicate that climate change may not only decrease precipitation, it may also increase the rates of evapotranspiration, and reduce the stream flows^57^. Under forthcoming warming, the decrease in both snowmelt and infiltration can decrease recharge of groundwater, leading to deeper water table and declined spring flows. The reason can be accredited to the decline in snowfall over the Kashmir valley during the past few decades, which decreases the snowmelt, hence infiltration and recharge of groundwater. Also, due to increasing temperatures and CO_2_ fertilization, the vegetation grows more rapidly leading to increase in transpiration during the growing season and reduces the recharge of groundwater into aquifers. It is pertinent to mention here that certain mechanisms upsetting the recharge of spring water in the Kashmir valley are already happening. For example, changes in magnitude and timing of snowmelt have been reported from Kashmir valley^58^ and likely influence the recharge of spring water.

Although once known as a state having surplus water with low population densities, Kashmir valley has recently seen a significant increase in population and water demand^59^. Freshwater resources like springs were used by people to satisfy their water demand but from past few years people are facing severe water shortages despite the fact that government has improvised water supplies^32^. The combined effect of climate change and population growth is likely to challenge the future freshwater availability^60^. Population growth has led to an overall increase in water demand (per capita increase) and pressure on freshwater resources. According to the Census of India^61^, the current population in Kashmir valley is 6.89 million and the projected population by the year 2051 is 14.41 million (Supplementary Figure S1). Based on per capita per day consumption (135 L/day) estimates of Public Health Engineering Department^62^, future demand was forecasted. The current total domestic demand is estimated to be 235 billion liters/year and is projected to reach 850 billion liters/year by 2050 (Supplementary Figure S2). With growing water scarcity, exacerbated by climate change and population growth, springs are likely to play a vital role in meeting the domestic water demand in future. Proper management and conservation of spring water resource in the face of climate change require knowledge of their potential, demand, availability, quality and recharge.

## Conclusion

Variation in the in-situ hydrogeochemical characteristics of 258 springs spread across the Kashmir valley were studied. The type of hydro-chemical facies (water type) was characterized based on relative abundance of major cations (Ca^2+^ and Mg^2+^, Na^+^ and K^+^) and anions (HCO_3_^−^, SO_4_^2−^ and Cl^−^). Piper trilinear diagram used to study the hydro-chemical facies (water types) based on relative abundance of ions indicate Ca–Mg–HCO_3_ dominating the major spring waters suggesting springs are fed mostly by shallow fresh groundwater. The Gibbs plot shows that the water quality of springs in Kashmir valley is mainly influenced by rock-water interaction factor, and to the minor extent by precipitation factor. The water quality index indicated that 87% of the springs have excellent to good water which means that most of the springs are a safe source of fresh drinking water for the population in the study area. The present study highly recommends the continuous monitoring and assessment of spring water sources in Kashmir valley for proper management of these indispensable natural resources in the context of climate change.

## Materials and methods

### Study area

**Figure 5:**
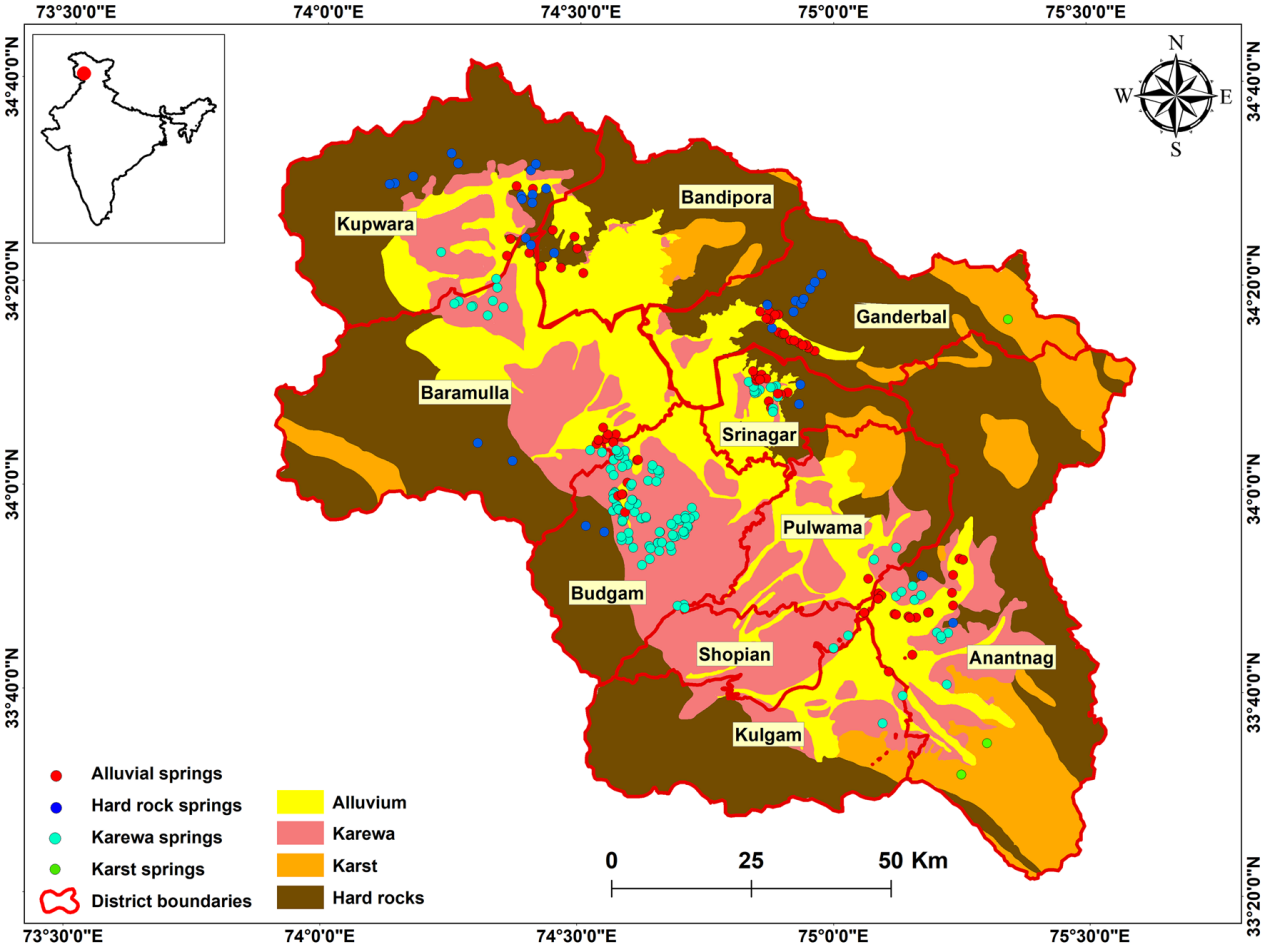
Location of freshwater springs and geological map (Modified after Dar and Zeedan, 2020)^66^ of the Kashmir valley. The figure was generated in ArcGIS version 10.4.1. (https://www.esri.com/en-us/arcgis/products/arcgis-pro/).

Kashmir valley covering an area of 15,948 km^2^, is situated on the northwestern part of the IHR, between 36° 58′–32° 17′ N latitudes and 80° 30′–73° 26′ E longitudes (Fig. 5). The elevation of the valley varies from 1080 to 5260 m above mean sea level. The valley has a distinctive continental climate, with a marked seasonality characteristic of the sub-continent of India^63^. Based on the overall physical characteristics of local weather, the valley has four weather seasons spring, summer, autumn, and winter. The mean annual precipitation of the valley is ~ 1240 mm year^−1^, and the monthly temperature varies from − 5 °C to more than 30 °C. The Kashmir Himalayan region supports a rich floral and faunal diversity in association with its unique geographical position, temperate climate and varied terrain. The abundance and diversity of water resources and associated biodiversity in the Kashmir valley, including glaciers, lakes, rivers, streams, springs, ponds, and wetlands, is unmatched in the entire Himalayan region.

In the Kashmir valley, freshwater springs occur widely, including in both high-altitude areas and plains (Fig. 5). Across the valley, numerous springs provide freshwater year-round. In Kashmir Himalaya, the human population is experiencing a massive growth rate and providing sufficient potable water is a challenge for water resource managers. As per Census, 2011^61^, Kashmir valley has a population of 6,888,475 persons which is projected to reach 14.41 million by the year 2051. This large human population increase, together with massive urbanization has largely damaged the fragile ecosystems of the Kashmir Himalayan region with severe consequences for the long-term sustainability of water resources. A large human population has led to an increasing demand for water supplies, and as a result, many areas are facing the risk of water crisis, including the diminishing and drying of springs.

### Regional geology

Geologically, the Kashmir valley has rocks of all ages, from recent alluvium to the old Archean, and preserves a successive record of volcanism, tectonics, and sedimentation that accompanied the Himalayan orogeny^64^. Bounded by the Greater Himalayan Range to the north-east and Pir-Panjal Range to the south-west, the valley has a record of tectonic activity and the subsequent evolution of landscapes in the form of several tectonic and sedimentary structures. Quarternary (Karewa), Triassic (carbonate), Palaeozoic (silicate and carbonate), and Recent (alluvium) rock deposits are the main geographical components in the Kashmir valley^65^ (Fig. 5). Tectonic-geomorphological studies in the Kashmir valley support the existence of a vast lake (often called Karewa Lake) that once occurred in the present Kashmir valley, as indicated by extensive lacustrine deposits from the Udars or Karewas plateau^66^. The sedimentation in Lake Karewa occurred during two phases (Lower and Upper Karewa) in the Pliocene epoch, as indicated by the Hirpur and Nagum formations, respectively^67^. The Karewa region is 12–25 km wide in the southwest, and extends about 80 km from south (Shopian) to north (Baramulla). In Kashmir valley, Karst is widespread due to the varied distribution of carbonate rocks, mainly towards the southern frontier of the valley. The Kashmir Valley is characterized by diverse karst features, including not only diverse cold and warm springs, but also caverns, conduits, shafts, sinkholes, pits and karren fields that are most established in Triassic limestone located in the southern portion of the valley.

### Water quality evaluation

#### Sample collection and analysis

Evaluating the quality of spring water is an imperative aspect in determining its fitness for drinking purposes. The water samples were collected from 258 springs well distributed over the entire Kashmir valley. The standard methodology was followed for collection of water samples^68^. The spring water samples were collected in a pre-cleaned high-density polythene sample bottle of 1-L capacity. After labeling, the samples were transported to aquatic laboratory in the Department of Environmental Science, University of Kashmir. Prior to the analysis, the samples were stored at a temperature below 4 °C. All physiochemical and biological parameters such as bicarbonate alkalinity, total hardness, calcium, magnesium, chloride, nitrate, sulfate, iron, total phosphorus, sodium, potassium, and coliform were analyzed following the standard methods recommended by the American Public Health Association^68^.

Physicochemical parameters such as hydrogen ion concentration (pH), electrical conductivity (EC), total dissolved solids (TDS), and Salinity were measured on-site in the field by dipping portable digital probe (PCS TESTR 35). Bicarbonate alkalinity was estimated against titration with HCl following the potentiometric titration. Total hardness, calcium, and magnesium were estimated using the EDTA titrimetric method. Chloride was estimated by titration against AgNO_3_ following the argentometric method. Nitrate was estimated using salicylate method. Sulfate was estimated using turbidimetric method. Iron was estimated by phenanthroline method. Total phosphorus was estimated using ascorbic acid method. The parameters like nitrate, iron, sulfate, and total phosphorus were estimated at their specific wavelengths using US made Thermo Scientific (Evolution 220) UV–Visible Spectrophotometer. Sodium and potassium were estimated following flame emission photometry method using Flame Photometer (SYSTRONICS 130). Coliform was determined by most probable number (MPN) using a multiple series of Durham tubes involving the presumptive, confirmed, and completed tests. The various physical, chemical, and biological parameters of spring waters were compared with drinking water quality standards set by WHO^37^ for their suitability for drinking purposes.

#### Ion error

The physicochemical analysis of water quality parameters in the laboratory involved a first step of evaluating the quality of data. This was accomplished by calculating the balance of positive and negative ions. The level of error was calculated using the following formula

$$E_{ib} = \frac{{\left( {\sum {cations - \sum {anions} } } \right)}}{{\left( {\sum {cations - \sum {anions} } } \right)}} \times 100$$

where E_ib_ is the error of ion balance. An error of up to ± 5% was considered as tolerable.

#### Spatial distribution maps

The coordinates of the sampling sites were recorded using a hand-held Global Positioning System (GPS), Garmin ETREX having an accuracy of ± 3 m. The geographical coordinates recorded at different springs were imported into a GIS platform. The GIS-based analysis of spatial behavior of the water quality in the study area was accomplished with the aid of the spatial analyst module and Natural Neighbor interpolation technique^69^ in ArcGIS version 10.4.1. (https://www.esri.com/en-us/arcgis/products/arcgis-pro/).

#### Water quality index

WQI has been widely used throughout the world to assess the quality of water for drinking purposes^70^. The water quality parameters were assigned different weights from 1 to 5 based on their critical health effects. The highest weight of 5 was allotted to parameters such as NO_3_ and Fe, due to their foremost importance in the evaluation of water quality, and least value of 1 was given to Na^+^, and K^+^. The water quality index was computed by the following equations:

1 $$W_{i} = \frac{{w_{i} }}{{\sum\limits_{i = 1}^{n} {w_{i} } }}$$

where *W*_*i*_ is the relative weight, *w*_*i*_ is the weight of each water quality parameter, and *n* is the number of parameters. Then, for each parameter, a quality rating was determined as follows:

2 $$Q_{i} = \frac{{C_{i} }}{{S_{i} }} \times 100$$

where *Q*_*i*_ represents the quality rating*, C*_*i*_ is the concentration of each water quality parameter, *Si* is the recommended standard value for each chemical parameter. Thereafter, to calculate WQI, the first sub-index (*S*_*i*_) was determined as:

3 $$S_{i} = W_{i} \times Q_{i}$$

where *S*_*i*_ symbolizes the sub-index of the ith parameter, and *W*_*i*_ and *Q*_*i*_ indicate the relative weight and quality rating of the ith parameter, respectively.

4 $$SWQI = \sum\limits_{i = 0}^{n} {S_{i} }$$

#### Principal components analysis

PCA converts various measured interconnected parameters into few orthogonal (uncorrelated) parameters known as principal components (PCs)^71,72^. The technique works with a relationship matrix and thus imitates the statistical relationships between parameters. Although the measured physicochemical water quality parameters that are evaluated are correlated, the calculated parameters (PCs) are uncorrelated and are obtained as a linear combination of the observable water quality parameters. The correlation coefficients obtained between the original parameters and PCs are the factor loadings, which quantify the weights of influence of each original variable on each PC. The PC can be expressed as:

5 $$z_{ij} = a_{i1} x_{1j} + a_{i2} x_{2j} + \cdots + a_{im} x_{mj}$$

where ‘z’ is the component score, ‘a’ the component loading, ‘x’ the measured value of parameter, ‘i’ the component number, ‘j’ the sample number and ‘m’ the total number of parameters. PCA was carried out with “FactoMineR” and “Factoextra” packages using R programming for statistical computing (https://cran.r-project.org/bin/windows/base/)^73^.

#### Hydro-chemical facies

Two hundred fifty-eight (258) springs selected for the study were categorized into four different classes: Alluvial, Karst, Karewa, and Hard rock springs based on geological and lithological features.

#### Piper diagram

For the identification of water types, the chemical analysis data of the spring water samples were plotted on a Piper diagram, using R programming for statistical computing (https://cran.r-project.org/bin/windows/base/)^73^. The piper trilinear diagram comprising of two ternary diagrams, lower left indicating cations and lower right indicating anions, and central diamond plot representing the matrix transformation of lower left and right ternary diagrams.

#### Gibbs diagram

Gibbs^74^ proposed two plots to recognize the natural mechanisms of surface water chemistry. These diagrams have been widely used to study the main mechanisms governing the chemistry of groundwater. Gibbs diagram was generated using MS Excel 2016 (https://support.microsoft.com/en-us/office). Gibbs diagrams depend on two ratios which are calculated by the succeeding equations

6 $$Gibbs\; ratio{\text{-}}I = \frac{{Cl^{ - } }}{{Cl^{ - } + HCO_{3}^{ - } }}$$

7 $$Gibbs\; ratio{\text{-}}II = \frac{{Na^{ + } + K^{ + } }}{{Na^{ + } + K^{ + } + Ca^{2 + } }}$$

## Supplementary Information

Supplementary Information.

## Data Availability

All data generated or analyzed during this study are included in this published article and Supplementary material.
