# Supplementary material for: A critical appraisal of the status and hydrogeochemical characteristics of freshwater springs in Kashmir Valley
Source: Sci Rep. 2022 Apr 6;12:5817. doi: 10.1038/s41598-022-09906-2 (PMC8987061; doi:10.1038/s41598-022-09906-2)
Supplement: Supplementary file 1 — Supplementary Information. [file 41598_2022_9906_MOESM1_ESM.pdf]

A critical appraisal of the status and hydrogeochemical characteristics of freshwater springs in Kashmir Valley

Sami Ullah Bhat[1*], Shahid Ahmad Dar[1] and Aadil Hamid[1]

[1]Department of Environmental Science, University of Kashmir, 190006, J&K, India

List of Tables

Table S1 Classification of spring waters for drinking purposes based on TDS (Davis and DeWiest, 1966), Total hardness (Sawyer and McCarthy, 1967) and Total Phosphorus (People's Republic of China), Zuo et al. 2013.

S. No.	TDS mg/l	Classification	No. of samples
1.	<500	Desirable for drinking	218
2.	500-1000	Permissible for drinking	40
3.	1000-3000	Useful for irrigation	-
4.	>3000	Unfit for drinking and irrigation	-
	**Total Hardness mg/l**		
5.	<75	Soft	115
6.	75-150	Moderately hard	69
7.	150-300	Hard	64
8.	>300	Very hard	10
	**Total Phosphorus**		
9.	≤ 20-200	Used for drinking purposes, fishing and recreation	172
10.	≤ 300	Used for industry and irrigation	20
11.	≤ 400	Cannot be used by any sector	66

Table S2 WQI in the study area

S. No.	Water Quality	Range	No. of Samples in the study area
1.	Excellent	0 - 50	102
2.	Very good	>50 - 100	123
3.	Poor water	>100 - 200	13
4.	Very poor water	>200 - 300	4
5.	Water unsuitable for drinking purposes	>300	16

Table S3 Principal component loadings for water quality parameters for the entire dataset

Variables	PC 1	PC 2	PC 3	PC 4
pH	0.154	0.076	-0.518	0.184
EC	0.098	0.477	0.283	0.054
TDS	0.080	0.539	0.272	0.039
Salinity	0.15	0.535	0.055	0.093
TA	0.295	-0.234	0.411	-0.180
TH	0.397	-0.200	0.166	0.082
Ca	0.392	-0.085	0.127	0.172
Mg	0.411	0.005	-0.188	0.103
SO_4	0.287	0.137	-0.389	-0.184
Fe	0.094	-0.020	0.016	-0.678
NO_3-N	0.348	0.054	-0.321	0.027
Cl	0.370	-0.081	0.175	-0.238
TP	0.125	-0.154	0.083	0.266
Coliform	0.044	-0.180	0.178	0.502
Eigen values	4.31	2.610	1.300	1.140
% Variance	31.08	18.698	9.290	8.166
Cumulative % variance	31.08	49.782	59.073	67.24

List of Figures

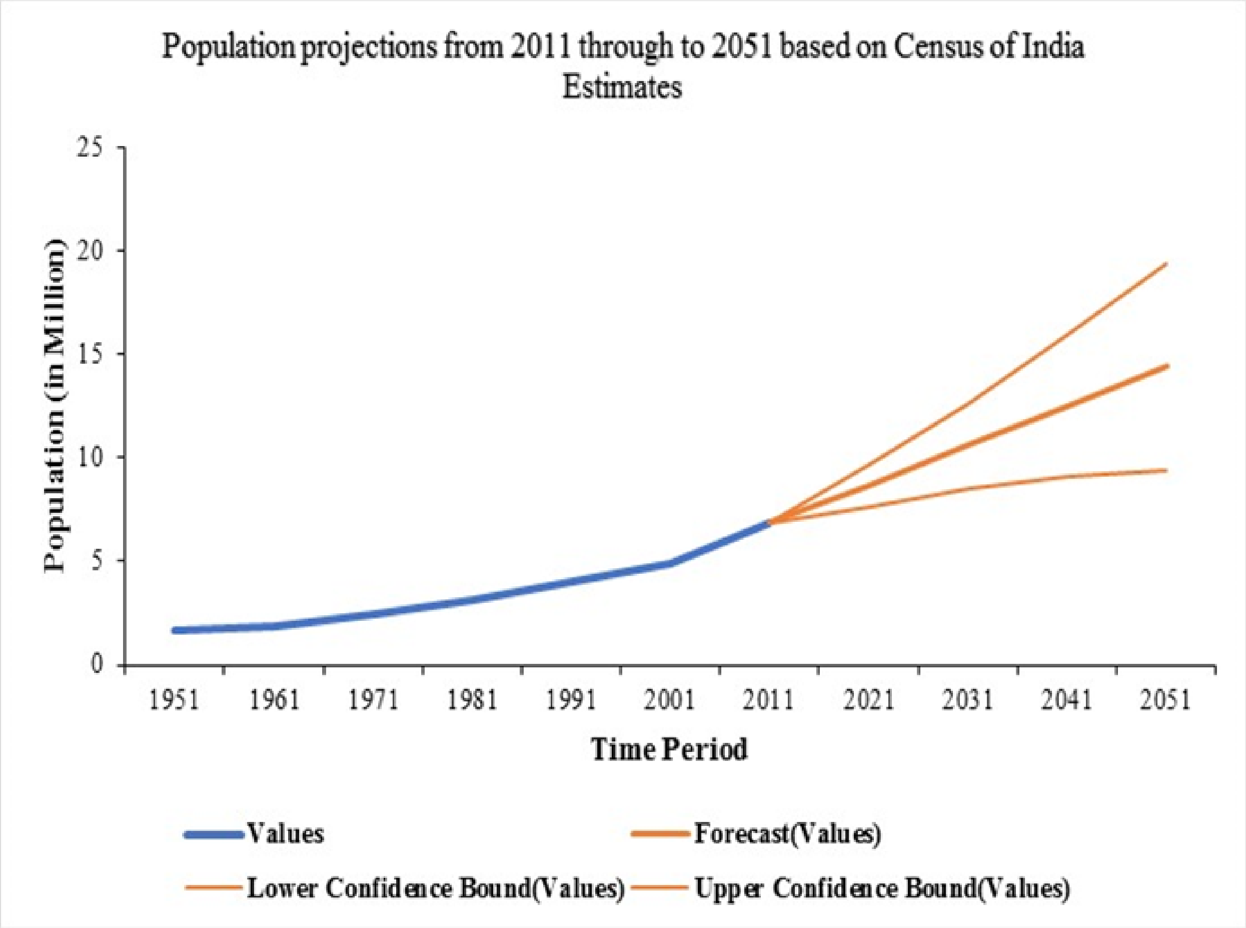

Figure S1 Population projection for Kashmir Valley from 2011 through 2051 based on Census of India estimates

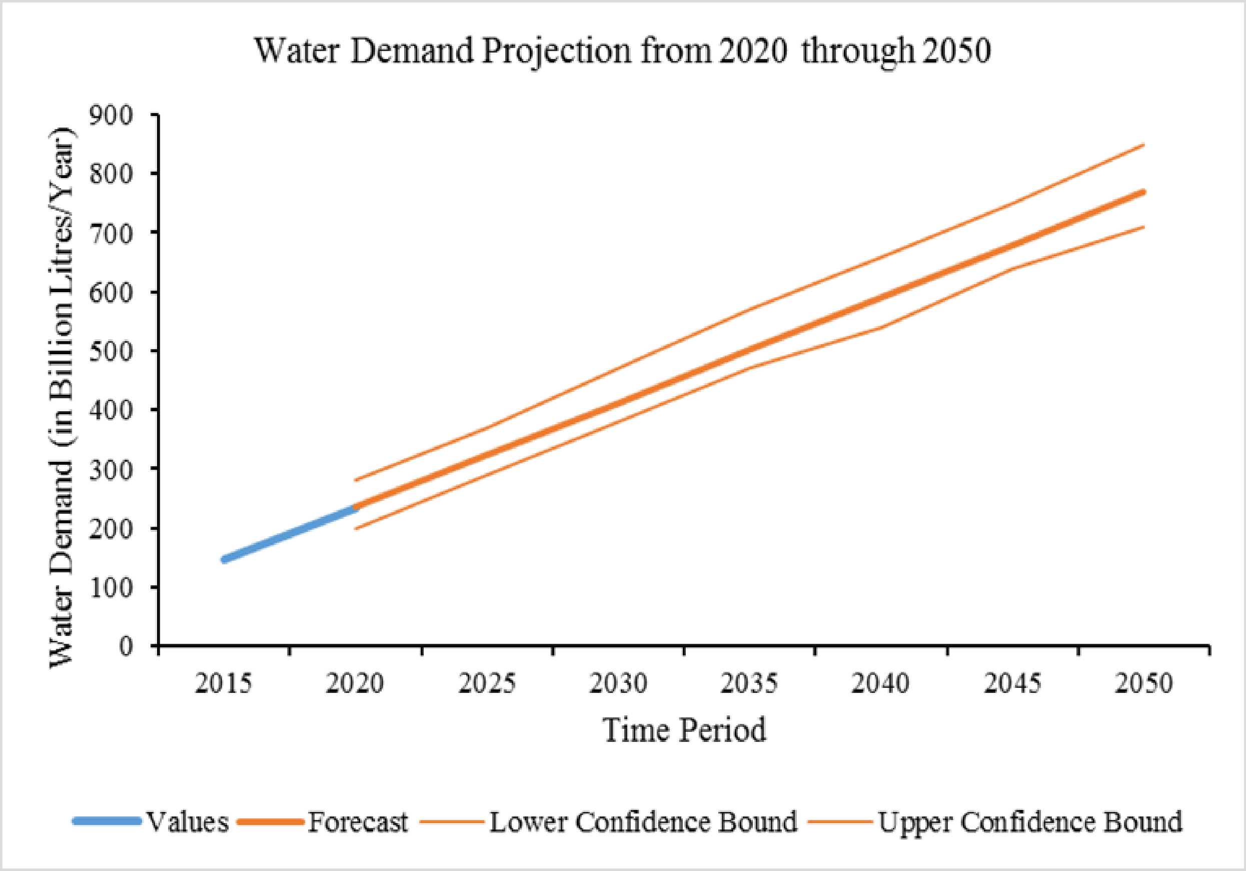

Figure S2 Total domestic water demand projection for Kashmir Valley from 2021 through 2050 based on PHE estimates
